# Correction to: Diacerein improves liver fibrosis, steatosis, and atherosclerosis in ApoE knockout mice

**DOI:** 10.1007/s00109-026-02674-w

**Published:** 2026-04-14

**Authors:** Jie-Eun Lee, Isom Jin, Jung-Jae Lee, Jisu Jung, Jee-In Lee, Bo-Rahm Kim, Tae Jung Oh, Yun Kyung Lee, Sung Hee Choi

**Affiliations:** 1 Department of Internal Medicine, CHA University Gangnam Medical Center, Seoul, Republic of Korea; 2 Department of Internal Medicine, Seoul National University Bundang Hospital, Seongnam, Republic of Korea; 3 Department of Tropical Medicine and Parasitology and Institute of Endemic Disease, Seoul National University College of Medicine, Seoul, Republic of Korea; 4 Department of Internal Medicine, College of Medicine, Seoul National University, Seoul, Republic of Korea

**Correction to: Journal of Molecular Medicine**

10.1007/s00109-026-02653-1

Figure 2B is missing in the image of Figure 2.
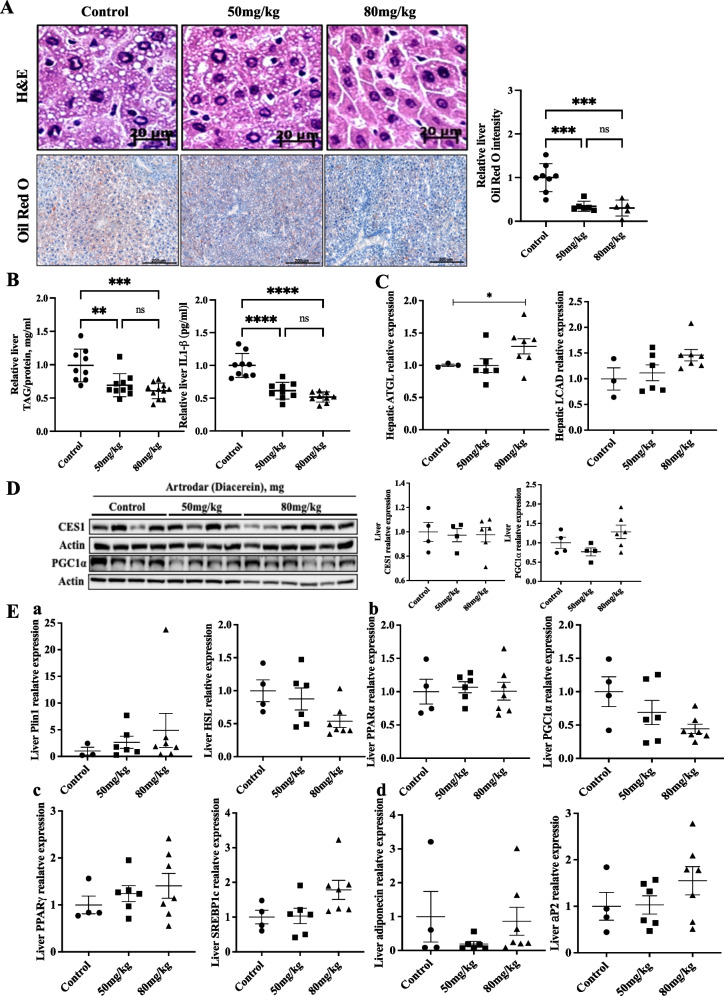


The Original article has been corrected.

